# Methodology and validation of a new tandem mass spectrometer method for the quantification of inorganic and organic ^18^O-phosphate species

**DOI:** 10.1371/journal.pone.0229172

**Published:** 2020-02-24

**Authors:** Aimée Schryer, Kris Bradshaw, Steven D. Siciliano

**Affiliations:** 1 Department of Soil Science, University of Saskatchewan, Saskatoon, Saskatchewan, Canada; 2 Federated Co-operatives Limited, Saskatoon, Saskatchewan, Canada; Sichuan Agricultural University, CHINA

## Abstract

Phosphorus (P) fertilizers are crucial to achieve peak productivity in agricultural systems. However, the fate of P fertilizers via microorganism incorporation and the exchange processes between soil pools is not well understood. ^18^Oxygen-labelled phosphate (^18^O- P_i_) can be tracked as it cycles through soil systems. Our study describes biological and geochemical P dynamics using a tandem mass spectrometry (MS/MS) method for the absolute quantification of ^18^O- P_i_. Soil microcosms underwent three treatments: (i) ^18^O- P_i_, (ii) unlabelled phosphate (^16^O- P_i_) or (iii) Milli-Q control, dissolved in a bio-stimulatory solution. During a 6-week series the microcosms were sampled to measure P by Hedley sequential fractionation and DNA extraction samples digested to 3′-deoxynucleoside 5′-monophosphates (dNMP). A MS/MS attached to a HPLC analyzed each P-species through collision-induced dissociation. The resin-extractable and bicarbonate ^18^O- P_i_ and ^16^O- P_i_ fractions displayed similar precipitation and adsorption-desorption trends. Biotic activity measured in the NaOH and dNMP fractions rapidly delabelled ^18^O- P_i_; however, the MS/MS measured some ^18^O that remained between the P backbone and deoxyribose sugars. After 6 weeks, the ^18^O- P_i_ had not reached the HCl soil pool, highlighting the long-term nature of P movement. Our methodology improves on previous isotopic tracking methods as endogenous P does not dilute the system, unlike ^32^P techniques, and measured total P is not a ratio, dissimilar from natural abundance techniques. Measuring ^18^O- P_i_ using MS/MS provides information to enhance land sustainability and stewardship practices regardless of soil type by understanding both the inorganic movement of P fertilizers and the dynamic P pool in microbial DNA.

## Introduction

Phosphorus (P) is an essential macronutrient, yet it is frequently the limiting factor for biological activity in soils worldwide [1]. Applying P-phosphate fertilizers increases soil sustainability, crop yields and promotes other biological processes, such as contaminant bioremediation [2,3]. The primary source available for plants and microbial communities is inorganic phosphate; consequently, extraction methods emphasize quantification of the inorganic phases to estimate P availability [4–7]. Despite the importance of inorganic P, organic P represents a significant portion of both total and bioavailable P within soils [8]. A large part of the organic P fraction is bound in microorganisms, primarily in nucleic acids, phospholipids, inositol phosphate, sugar phosphates, and as condensed P [5,6]. Nevertheless, the precise composition of organic P within soils is poorly understood [5]. Microorganisms mediate key processes within the biogeochemical P cycle, such as immobilization and mineralization, strongly influence P bioavailability for other biotic species [9]. While many techniques estimate the concentration of organic P, including fumigation for microbial biomass and sequential fractionation for total organic P, they cannot identify the chemical nature or the cycling dynamics of organic P [10].

To investigate organic P dynamics, investigators typically resort to isotopic methods such as the isotope dilution protocol, which uses ^32^P and ^33^P to determine soil organic P permutation and concentration dynamics [8,11]. This technique monitors the exchange between a known concentration of dosed ^32/33^P-phosphate fertilizers and endogenous ^31^P-phosphates in treated soils [12]. Work with ^32/33^P increased understanding of the P-cycle by assessing the sizes and rates of exchange of ^32/33^P between P pools and/or tracking fertilizer P fate from soil to biota, (e.g., plant and microbial communities) [4,13–16]. However, due to the radioactive nature of tracer ^32/33^P, current isotope dilution techniques are difficult to integrate with genomic pipelines. This includes difficultly in identifying what type of organic P is moving within biotic systems. The transmutation of ^32/33^P to daughter species ^32/33^sulfur produces an unstable coordination number and high vibrational energy [17]. Consequently, the half-lives of a radionucleotide is 5–20 times shorter than the radioisotope as the radionucleotides self-destructs [18]. Interactions with released energy or with any radiation-produced reactants (i.e., radicals) from labelled molecules causes damage to nearby biomolecules and nucleic acids [19]. Additionally, the half-lives of ^32^P and ^33^P (14.3 days and 24.4 days) restrict the length of studies due to self-radiolysis [4,18]. Equilibration times for the P species further complicates experimental design as a portion of mineral inorganic P is rapidly exchangeable with solution inorganic P [20]. For example, the isotopic equilibration rate between endogenous ^31^P and experimental ^32/33^P fertilizers requires three months or between six (^32^P) and three (^33^P) half-lives [21]. While ^32/33^P studies provide the basis for understanding both soil fertility and P cycling, a non-radioactive tracer is needed to complement current work into organic P dynamics.

Oxygen is an ideal stable isotope to discern the biogeochemical cycle of P. Oxygen has three stable isotopes while P only has one (^31^P) [1,22]. The natural abundance of ^18^O is 0.204% and the two additional neutrons allow the separation between labelled and unlabelled fractions during downstream genomic applications [23,24]. Only enzyme mediated biological activity breaks the O-P bond under environmentally relevant conditions as it is stable under ambient temperatures and in abiotic environments [25–27]. The ubiquitous intracellular enzyme pyrophosphatase exchanges ^18^O and ^16^O present in cellular fluids and water until it reaches equilibrium [28]. Moreover, the enzyme is stable following cell lysis and will exchange atoms outside the cell [28]. Melby et al. [29] described that the half-life of ^18^O-Orthophosphate (P_i_) as 15 to 22 days in non-sterile soils and greater than 50 days in sterile soils. One option to track P trends is to measure the stable isotope ratio of oxygen(δ^18^O_p_) by isotope ratio mass spectrometry [IRMS, e.g. 22–24]. Samples are not directly analyzed. Alternatively, P_i_ undergoes processing to silver phosphate (Ag_3_PO_4_) with subsequent purification steps to minimize contamination from other O-isotope sources, such as oxyanions [30]. The samples are pyrolyzed in a thermochemolysis/elemental analyzer at 1460°C, converted to C^18^O and C^16^O gas, measured by IRMS and described using the following equation [30] [31];

$$\delta^{18}O_{P}=\left(\frac{R_{sample}}{R_{standard}}-1\right)*1,000$$

However, δO vary by soils, sites and environments; this variation coupled with instrument sensitivity precludes the use of δO as a proxy for cycling of organic P [32–34]. In contrast, the use of enriched ^18^O-P is well suited for stable isotope probing (SIP) [32,35]. Stable isotope probing tracks isotopically labelled substrates to determine nutrient movement within abiotic systems and organisms while concurrently identifying active microbial populations and biological processes [24,36,37]. The methodology can also follow both inorganic P pools within the environment using ^18^O enriched fertilizers [38]. Thus, SIP experiments in soils need to balance the time required for refractory P-pools to be labelled with the decay in signature of the original source of ^18^O-P by microbial communities[32,33]. Mass spectrometry is capable of following the unpredictable biotic and inorganic ^18^O-SIP signature within soil systems due to its’ sensitivity, accuracy, and its capability to concurrently measure analytes from a wide range of masses [39]. A MS instrument comprises of three elements: an ion source, mass analyzer and a detector [40,41]. The ion source produces charged gas phase ions from either liquid or solid phase samples [39]. Analyzers sort ions by mass using electromagnetic fields, thereby determining the isotopic composition of compounds [42]. To increase the selectivity of the analysis, the multiple step selection method known as tandem mass spectrometry (MS/MS) isolates precursor ions and produces known product ions [43]. Once through the mass analyzer, the detector performs both qualitative and quantitative analysis of the gas phase species through measuring the mass-to-charge (m/z) ratios and abundances [41,44]. Both the m/z accuracy and sensitivity for trace samples signifies that mass spectrometry is ideal to examine ^18^O- P_i_ fertilizer movement and biotic exchange effects overtime; however, MS investigations into P cycling in soils are limited.

In this study, we conducted experiments to validate both MS and the use of ^18^O labelled P_i_ to track the movement and dynamics of P in inorganic and organic pools. We hypothesize that the combination of labelled ^18^O- P_i_ SIP with high-performance liquid chromatography tandem mass spectrometry (HPLC-MS/MS) provides new opportunities to follow the fate of P fertilizers to better comprehend the P cycle in soils. This was completed in four steps. First, two mass spectrometer (MS) methods were created to quantify the concentration of unlabelled (^16^O)- and ^18^O- P_i_ and ^16^O- and ^18^O-3′-deoxynucleoside 5′-monophosphates (dNMP). Secondly, we compared concentration of resin-extractable ^16^O- and ^18^O- P_i_ using the SEAL segmented flow analyzer (AA3) and the HPLC-MS/MS method to determine whether the extraction matrices or instrumentation hindered the analysis of P_i_. Third, we doped soil with ^16^O- and ^18^O- P_i_ fertilizer in an ecologically relevant context to study P dynamics, ie. calcareous soil under anaerobic conditions, to both affirm the validity of the MS methods to track P and to view the differences in P movement in soils over a 6 week time series by sequential fractionation extraction. We used anaerobic conditions as our work focusses on P dynamics in polluted soils in which P is added to bio-stimulate remediation [3]. Fourth, we extracted DNA to measure the concentration of ^16^O- and ^18^O-dNMP between weeks to view changes in this significant portion of microbial organic P concentration and isotopic exchange over time. Finally, the sequential fractionation results were combined to create a mass balance of total P by isotopic composition to compare the recovery of each P_i_ species.

## Materials and methods

### Microcosm design

Soil samples were collected from Davidson (51°15'46.7"N, 105°59'36.9"W), Outlook (51°28'27.3"N, 107°06'04.6"W), and Allan (51°53’42.38”N, 106°03’22.02”W) in Saskatchewan, Canada. A total of 72 microcosms (3 treatments x 4 replicates x 6 time points) were created by homogenizing different quantities of soils from the three sites. Soil (10 g) was added to an acid bathed and autoclaved 30 mL serum bottle (Wheaton, Chicago, IL, USA). Each microcosm was filled with one of three treatments: (i) bio-stimulatory solution with either ^18^O- P_i_ or (ii) ^16^O- P_i_ as the P source or (iii) Milli-Q water only as a control. The ultra-purified Milli-Q water was obtained from an in-house purification system Milli-Q Direct 8/16 System (Millipore, Billerica, MA, USA). The bio-stimulatory solution comprised of 0.24 mM HNO_3_ [3.4 mg/L N], 0.24 mM Fe(III)NH_4_-citrate [13 mg/L Fe(III)], 22 mM SO_4_ [700 mg/L S]), and 0.1 mM P-species (3.1 mg/L P) in Milli-Q water at a circumneutral pH. Both ^16^O- and ^18^O- P_i_ were synthesized based on the procedure published by Melby et al. [45] using Milli-Q H_2_^16^O and H_2_^18^O (97% ^18^O; Millipore Sigma, Burlington, MA, USA) and Phosphorus Pentachloride (Millipore Sigma). The amount of labelling within each P source was checked by MS for isotopic purity. Each microcosm received 32 mL of the applicable solution to ensure complete saturation and was crimp sealed within an anaerobic chamber for minimal O_2_ conditions. The closed microcosms were mixed for 1 hour following assembly on a horizontal rotary shaker (150 rpm) and incubated at room temperature.

Microcosms were randomly assigned incubation time points: 1 week, 2 weeks, 3 weeks 4 weeks, 5 weeks, and 6 weeks following construction. Following incubation, microcosms were destructively sampled using vacuum filter units fitted with autoclaved 0.45 μm filter paper into acid bathed and autoclaved Büchner flasks to separate the soil and water solution. Soil samples were collected for sequential P extraction and microbial DNA. Aliquots of soil samples were ground to 0.85 mm [46]. Soil P fractions were extracted following the Hedley method developed by Tiessen and Moir [46] using resin anion exchange strips, followed by 0.5 M bicarbonate (pH = 8.5), 0.1 M NaOH, and 1.0 M HCl. For 0.5 M bicarbonate (7.0 g/30 mL, end pH = 3.4-3.7) and 0.1 M NaOH (1.5 g/30 mL, end pH = 5.2-5.4) extractions. AG 50W-X8 cation exchange resin beads (Bio-Rad Laboratories, Hercules, USA) were added to exchange sodium ions with protons to clean and acidify the sample. Microbial DNA was collected using a PowerSoil® DNA isolation kit (MoBio Technologies, Vancouver, BC, Canada) and eluted at 40 μL, followed by quantification using a Qubit 2.0 fluorometer (Invitrogen, Carlsbad, CA, USA).

### Mass spectrometric optimization of ^16^O- and ^18^O-P_i_ and ^16^O-dNMP standards

The collision-induced dissociation (CID) tandem mass spectrometric (MS/MS) optimization and analysis of ^16^O- P_i_, ^18^O- P_i_ and ^16^O-dNMP were conducted using a AB Sciex 4000 QTRAP® mass spectrometer (AB Sciex, Concord, ON, Canada) attached to an Agilent 1260 Infinity II HPLC System (Agilent Technologies, Santa Clara, CA, USA). The MS, a hybrid triple quadrupole–linear ion trap mass spectrometer (QqQ-LIT), is equipped with a Turbo V^™^ Ion Spray electrospray ionization (ESI) source with nitrogen utilized as the collision gas. The HPLC is composed of a binary pump with an autosampler that has temperature control. Both P_i_ and dNMP optimization were conducted in negative ion mode, where the collisional energy varied between -20.0 and -5.0 V, whereas the declustering potential remained fixed at -40 V. An integrated syringe pump (Harvard Apparatus, MA, USA) infused sample aliquots into the mass spectrometer at a rate of 10 μL /min through a Turbo Ionspray Source, where the needle voltage was -4500 V. Nitrogen was used both as the drying gas and ESI nebulizing gas. The fractionation pattern, product ions and MS conditions for ^16^O- P_i_ and ^18^O- P_i_ (Table 1) were identified. Similarly, the fractionation pattern, product ions and MS conditions for each ^16^O-dNMP (Table 2) were deduced.

**Table 1:** The mass spectrometry parameters for quantification of ^16^O- and ^18^O-P_i_.

ID	Q1	Q3	DP	EP	CE	CXP
	————amu———		—————————volts—————————			
^16^O- P_i_	96.9	78.8	-55	-10	-22	-5
		63.0	-55	-10	-62	-3
^18^O- P_i_	104.9	84.9	-55	-10	-20	-5
		67.0	-55	-10	-64	-1

Q1, quantifier precursor ion; Q3, quantifier product ions; DP, declustering potential; EP, entrance potential; CE, collision energy; CXP, Collision cell exit potential; amu, atomic mass unit (Daltons); ^16^O- P_i_; ^16^O-orthophosphate; ^18^O- P_i_, ^18^O-orthophosphate.

**Table 2:** The mass spectrometry parameters for quantification of ^16^O- and ^18^O-dNMP, and internal standard dIMP.

ID	Retention Time	Q1	Q3	DP	EP	CE	CXP
	min	———amu———		————————volts————————			
^16^O-dAMP	12.94	329.9	78.9	-105	-10	-58	-5
			134.1	-105	-10	-36	-9
^18^O-dAMP		332.2	80.9	-105	-10	-58	-5
			134.1	-105	-10	-36	-9
		334.1	82.9	-105	-10	-58	-5
			134.1	-105	-10	-36	-9
		336.1	84.9	-105	-10	-58	-5
			134.1	-105	-10	-36	-9
		338.1	84.9	-105	-10	-58	-5
			134.1	-105	-10	-36	-9
^16^O-dCMP	12.58	306.0	78.9	-85	-10	-58	-3
			110.1	-85	-10	-32	-7
^18^O-dCMP		308.1	80.9	-85	-10	-58	-3
			110.1	-85	-10	-32	-7
		310.1	82.9	-85	-10	-58	-3
			110.1	-85	-10	-32	-7
		312.1	84.9	-85	-10	-58	-3
			110.1	-85	-10	-32	-7
		314.1	84.9	-85	-10	-58	-3
			110.1	-85	-10	-32	-7
^16^O-dGMP	12.86	346.0	78.8	-90	-10	-66	-3
			150.1	-90	-10	-36	-11
^18^O-dGMP		348.1	80.9	-90	-10	-66	-3
			150.1	-90	-10	-36	-11
		350.1	82.9	-90	-10	-66	-3
			150.1	-90	-10	-36	-11
		352.1	84.9	-90	-10	-66	-3
			150.1	-90	-10	-36	-11
		354.1	84.9	-90	-10	-66	-3
			150.1	-90	-10	-36	-11
^16^O-dTMP	12.92	321.0	78.8	-70	-10	-78	-13
			124.8	-70	-10	-34	-13
^18^O-dTMP		323.1	80.9	-90	-10	-66	-3
			124.8	-70	-10	-34	-13
		325.1	82.9	-90	-10	-66	-3
			124.8	-70	-10	-34	-13
		327.1	84.9	-90	-10	-66	-3
			124.8	-70	-10	-34	-13
		329.1	84.9	-90	-10	-66	-3
			124.8	-70	-10	-34	-13
^16^O-dIMP	12.76	331.0	134.8	-85	-10	-34	-11
			194.9	-85	-10	-24	-5

Q1, quantifier precursor ion; Q3, quantifier product ions; DP, declustering potential; EP, entrance potential; CE, collision energy; CXP, Collision cell exit potential; amu, atomic mass unit (Daltons); dAMP, Deoxyadenosine monophosphate; dCMP, Deoxycystidine monophosphate; dGMP, Deoxyguanosine monophosphate; dTMP, Deoxythymidine monophosphate; dIMP, Deoxyidenosine monophosphate.

### Quantification of ^16^O- and ^18^O-P_i_

The concentration of ^16^O- and ^18^O- P_i_ following sequential P extraction was performed by direct infusion analysis on the 4000 QTRAP. The HPLC-MS/MS calibrations curves were produced in their respective sequential fractionation matrices from synthesized ^16^O- and ^18^O- P_i_ stocks following quantification on the SEAL segmented flow analyzer (AA3; Seal Analytical, Mequon, WI, USA). The optimized chromatographic and instrumental parameters for ^16^O- and ^18^O- P_i_ quantification on the HPLC-MS/MS are in S1 Table. The quality assurance (QA)/quality control (QC) for the method included: duplicates; spikes; and low, medium and high QC concentrations from the calibration curve in order to determine accuracy and any variation occurring intra- and inter-day. The concentration of the P_i_ in mg/L was determined by reporting the chromatographic peak areas of the samples versus standard solution concentrations using AB Sciex Analyst® Software version 1.6.2 (SCIEX. 2013. Analyst 1.6.2 Software Installation Guide. Framingham, MA, USA). The concentration of P_i_ was converted to mg/g dry soil by multiplying by the extraction volume and dividing by the mass of dry soil.

### Comparing instruments for the quantification of available ^16^O- and ^18^O-P_i_

The resin anion exchange strips extracted P_i_ was measured on both the AA3 and direct infusion analysis on the 4000 QTRAP HPLC-MS/MS. The AA3 calibration curve was produced from 1000 mg/L stock P solution (Cole-Parmer, Vernon Hills, IL, USA). The QA/QC for the AA3 included: duplicates, blanks, and method spikes.

### Digestion of DNA to dNMPs

Two enzymes were used to isolate dNMP from double stranded DNA following the method published by Bochkov et al. [47]. The double stranded DNA was combined with 2 μL DNAse I (1 unit (U)/μL, ThermoFisher, Waltham, MA, USA) and buffer and heated at 37°C for 15 minutes (min). Then 1 μL Nuclease S1 (100 U/μL, Promega, Madison WI, USA) and buffer was added and the solution was heated at 37°C for 15 min to release the dNMP (3′-deoxyadenosine 5′-monophosphate [dAMP], 3′-deoxythymidine 5′-monophosphate [dTMP], 3′-deoxycytidine 5′-monophosphate [dCMP] and 3′-deoxyguanosine 5′-monophosphate [dGMP]).

### Mass spectrometric analysis of ^16^O- and ^18^O-dNMP

Quantification of ^16^O- and ^18^O-dNMP species was completed using a calibration curve of ^16^O-dCMP (≥95.0%), ^16^O-dAMP (98-100%), ^16^O-dGMP (≥99%), and ^16^O-dTMP (≥99%) standards, all purchased from Millipore Sigma. The internal standard was deoxyinosine monophosphate (dIMP, Millipore Sigma), a structural analogue of the dNMP species. The chromatographic conditions and instrument parameters for dNMP quantification are in S2 Table. The QA/QC included: duplicates; spikes; and low, medium and high QC concentrations of the calibration curve. The concentration of dNMPs in mg/g soil was determined by reporting the chromatographic peak areas of the samples versus standard solution concentrations using AB Sciex Analyst® Software version 1.6.2 and correcting by the mass of soil used for DNA extraction and the final volume of the extraction (60 μL). The concentration of DNA- P_i_ from dNMPs was measured by adding the total concentration of each dNMP in each sample, where unlabelled dNMPs possessed 0 atoms of ^18^O atoms in the dNMP and labelled dNMP represented dNMPs with 1, 2, 3, or 4 ^18^O atoms.

### Statistical analyses

Statistical analyses were completed using R v.3.5.1 (R Core Team, 2018). The lowest detectable concentration with a signal-to-noise ratio of 3 was designated as the limit of detection (LOD) for each species [48]. The lowest concentration in the calibration curve yielding precision and accuracy within ± 20% was defined as the lowest limit of quantification (LLOQ). These parameters were measured using AB Sciex Analyst® Software version 1.6.2.

## Results

### Comparing AA3 and 4000 QTRAP P_i_ concentrations

**Fig 1:**
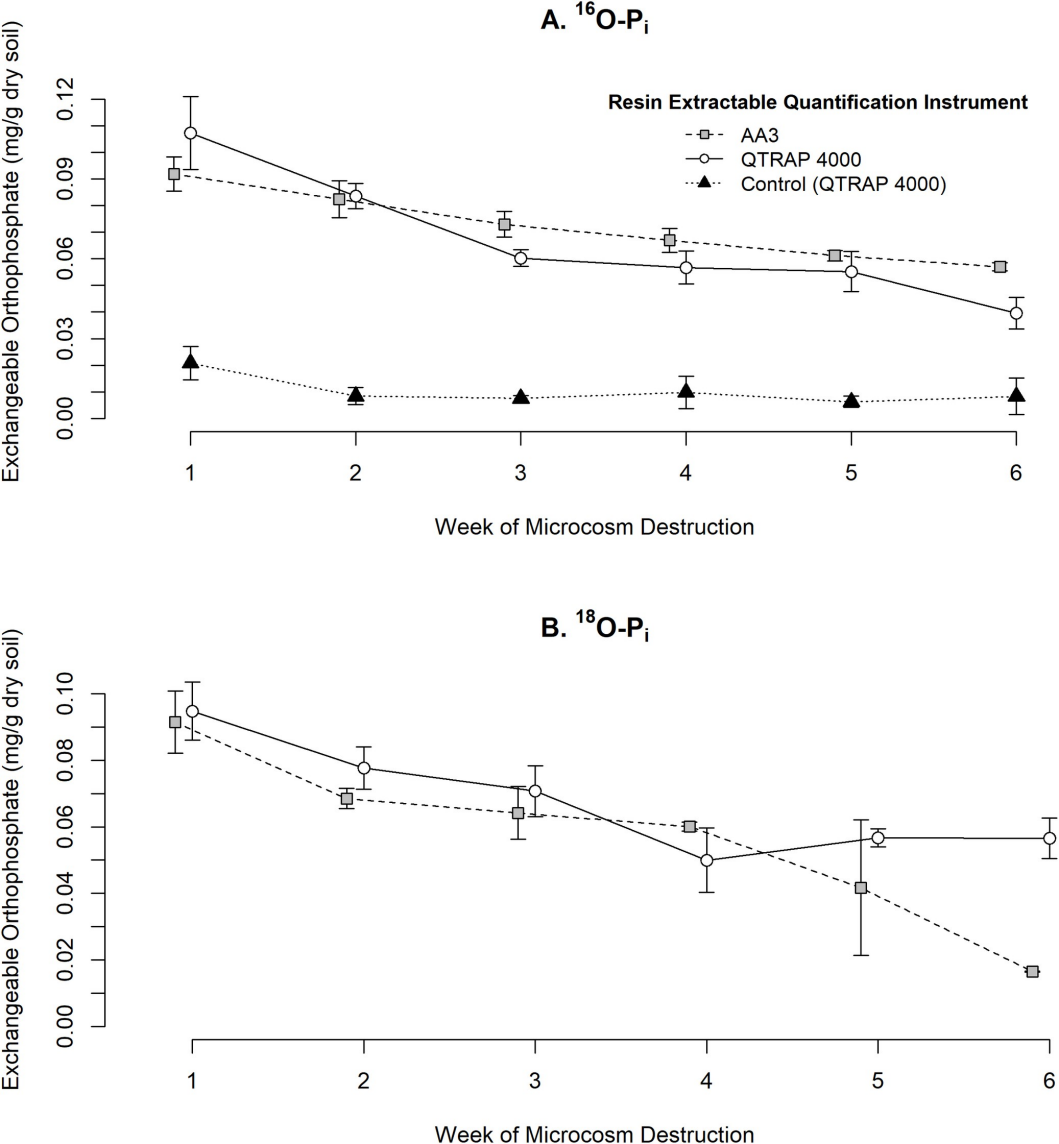
Concentration of resin-extractable P_i_ measured by AA3 and QTRAP 4000 versus week of microcosm destruction. At each time point, 0.5 g of soils were dried, sieved, and analyzed using strong anion resin strips. Each symbol represents the average of 4 microcosms ± standard errors of the estimates. Some symbols were offset on the x-axis to visualize the differences between treatment means. (A) The concentration of exchangeable ^16^O- P_i_ from ^16^O- P_i_ doped microcosms. (B) ^18^O- P_i_ concentrations from ^18^O- P_i_ doped microcosms.

During the time series, the AA3 and the 4000 QTRAP measured comparable concentrations of exchangeable ^16^O- P_i_ and ^18^O- P_i_ (Fig 1). Both instruments revealed a decrease in exchangeable ^16^O- P_i_ over the time series from ^16^O- P_i_ doped microcosms (Fig 1A). In comparison, there were no trends in the quantity of endogenous ^16^O- P_i_ in the control microcosms during the time series. Similar to ^16^O- P_i_, the concentration of exchangeable ^18^O- P_i_ decreased overtime on both instruments with the exception of weeks 5 and 6 (Fig 1B). There were no differences in the 4000 QTRAP measured P_i_ from weeks 4 to 6; however, the AA3 revealed a decrease in the quantity of P_i_ from week 4 to weeks 5 and 6. The poor similarity between instrumental analysis of weeks 5 and 6 is likely due to human error rather than differences between instruments. The ^18^O- P_i_ doped microcosms had a small invarying concentration of endogenous ^16^O- P_i_ and control microcosms had no ^18^O- P_i_ during the time series on the 4000 QTRAP. Generally, the 4000 QTRAP produced larger standard errors (SE) for each treatment in contrast to AA3 results. This may be because the AA3 is not as affected by the sample matrix in comparison to the MS. The LLOQ for both isotopic species on the AA3 was 0.1 mg/L. In contrast, the 4000 QTRAP LLOQ of ^16^O- P_i_ was 0.2 mg/L but the LOD was 0.1 mg/L. The LLOQ for^18^O- P_i_ was 0.1 mg/L and the LOD was 0.075 mg/L.

### Temporal ^16^O-P_i_ and ^18^O-P_i_ trends by treatment

**Fig 2:**
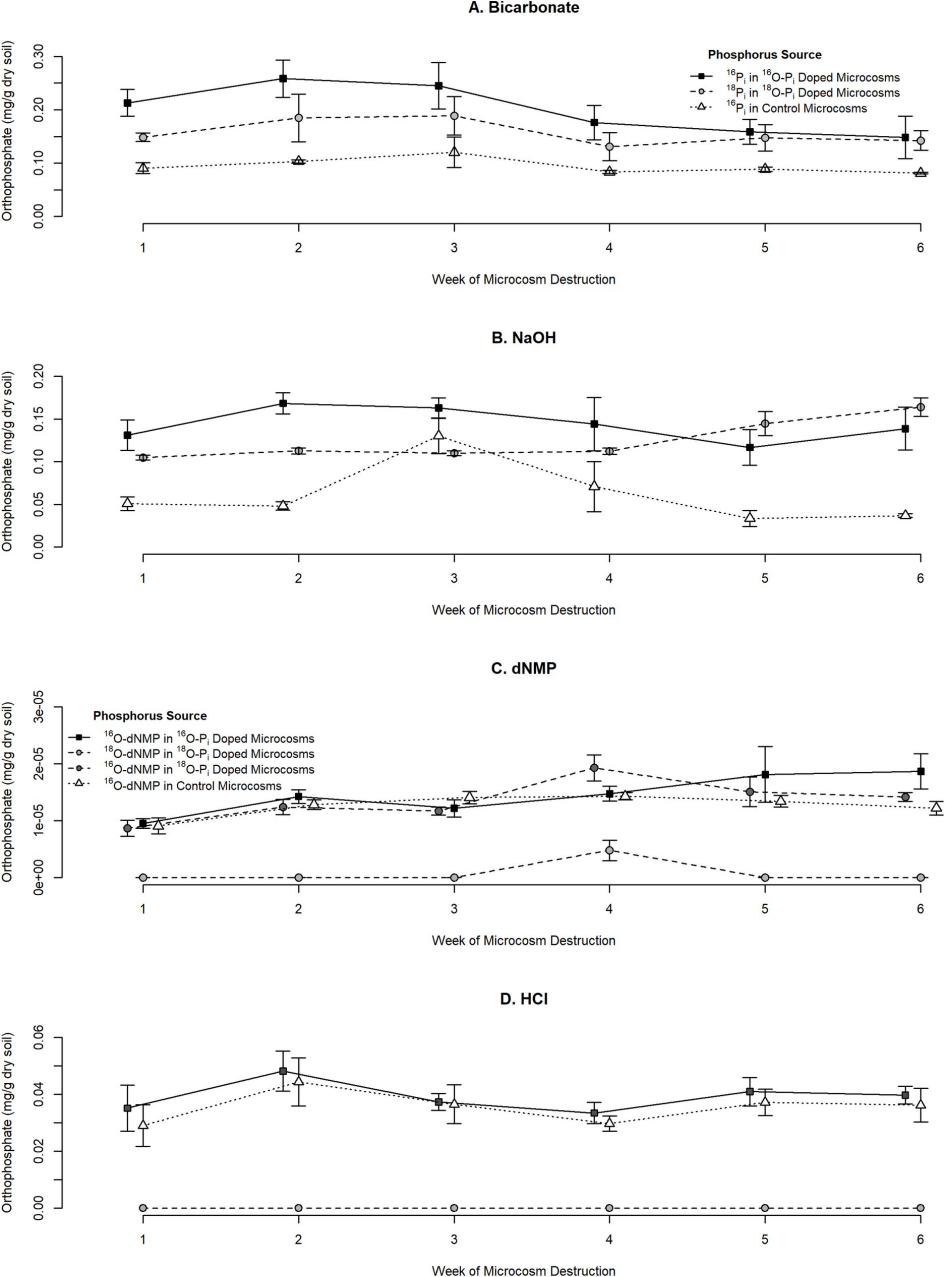
Temporal change in ^16^O-P_i_ and ^18^O-P_i_ in doped and control microcosms extracted by sequential fractionation. Each symbol represents the average concentration of 4 microcosms ± standard errors of the estimates. Sample means were off-set on the x-axes to see the treatment differences. (A) Concentration of P_i_ extracted by 0.5 M bicarbonate solution. (B) The concentrtation of P_i_ extracted by 0.1 M NaOH solution. (C) The concentration of dNMP. (D) The concentration of P_i_ extracted by 1.0 M HCl solution.

The average P_i_ concentration and temporal trends varied by sequential fractionation extraction method (Fig 2). There were shared characteristics between the trends of bicarbonate extracted ^16^O- P_i_ and ^18^O- P_i_ doped microcosms with no P_i_ concentration differences during the time series (Fig 2A). The quantity of ^16^O- P_i_ was greater than ^18^O- P_i_ as the instrument measures both doped and endogenous P_i_. The bicarbonate fraction represented the most dominant source of P_i_ within all experimental microcosms. However, this fraction also had the greatest variation, embodied by larger SE values. Endogenous ^16^O- P_i_ within control microcosms showed no trends during the time series. The LLOQ for both isotopic species was 0.3 mg/L and LOD was 0.2 mg/L.

The concentration of NaOH extracted ^16^O- P_i_ and ^18^O- P_i_ from doped microcosms was dissimilar during the time series (Fig 2B). The ^16^O- P_i_ doped microcosms showed little variation between weeks during the time series. In comparison, weeks 5 and 6 showed a noticeable increase in NaOH extracted ^18^O- P_i_. The decrease of resin-extractable and bicarbonate fraction of ^18^O- P_i_ suggests that labelled fertilizer shifted towards the NaOH pool. Once more, the concentration of ^16^O- P_i_ was greater than ^18^O- P_i_ as it characterized both doped and endogenous P_i_. The quantity of endogenous ^16^O- P_i_ within control microcosms increased until week 3 before rapidly decreasing at the end of week 6. Though the control microcosms did not receive a biostimulatory solution, the soil may have contained a small amount of endogenous nutrients that stimulated microbial communities. The LLOQ for both isotopic species was 0.2 mg/L and the LOD was 0.1 mg/L.

The first three weeks of the time series showed no differences between the treatments. Subsequently, treatments varied during week 4 (Fig 2C). The dNMP in ^16^O- P_i_ doped microcosms increased until week 5, signifying a potential stall in the microbial growth. Within DNA ^18^O concentrations were low. Specifically, the labelled portion of the dNMP molecules originated from the phosphodiester backbone, where a single ^18^O atom was present on the product ion. The peak of ^18^O-labelled DNA quantified on week 4 corresponded to the highest ^16^O-labelled DNA concentration in the same microcosm. The control microcosms did not show variation between weeks. The LLOQ for both isotopic species was 0.01 mg/L and the LOD was 0.0055 mg/L.

No apparent trends were present from the 1.0 M HCl extracted P_i_ from all microcosms during the time series (Fig 2D). Specifically, the concentration of ^16^O- P_i_ from ^16^O- P_i_ doped microcosms and from the control microcosms strongly correlate, demonstrating no variances during the time series. The HCl-extracted fraction from ^18^O- P_i_ microcosms measured no labelled species. This signifies that measuring isotopically labelled species within recalcitrant P fractions of soil requires a longer time series than provided. The LLOQ for both isotopic species was 0.2 mg/L and the LOD was 0.1 mg/L.

### P mass balance

**Fig 3:**
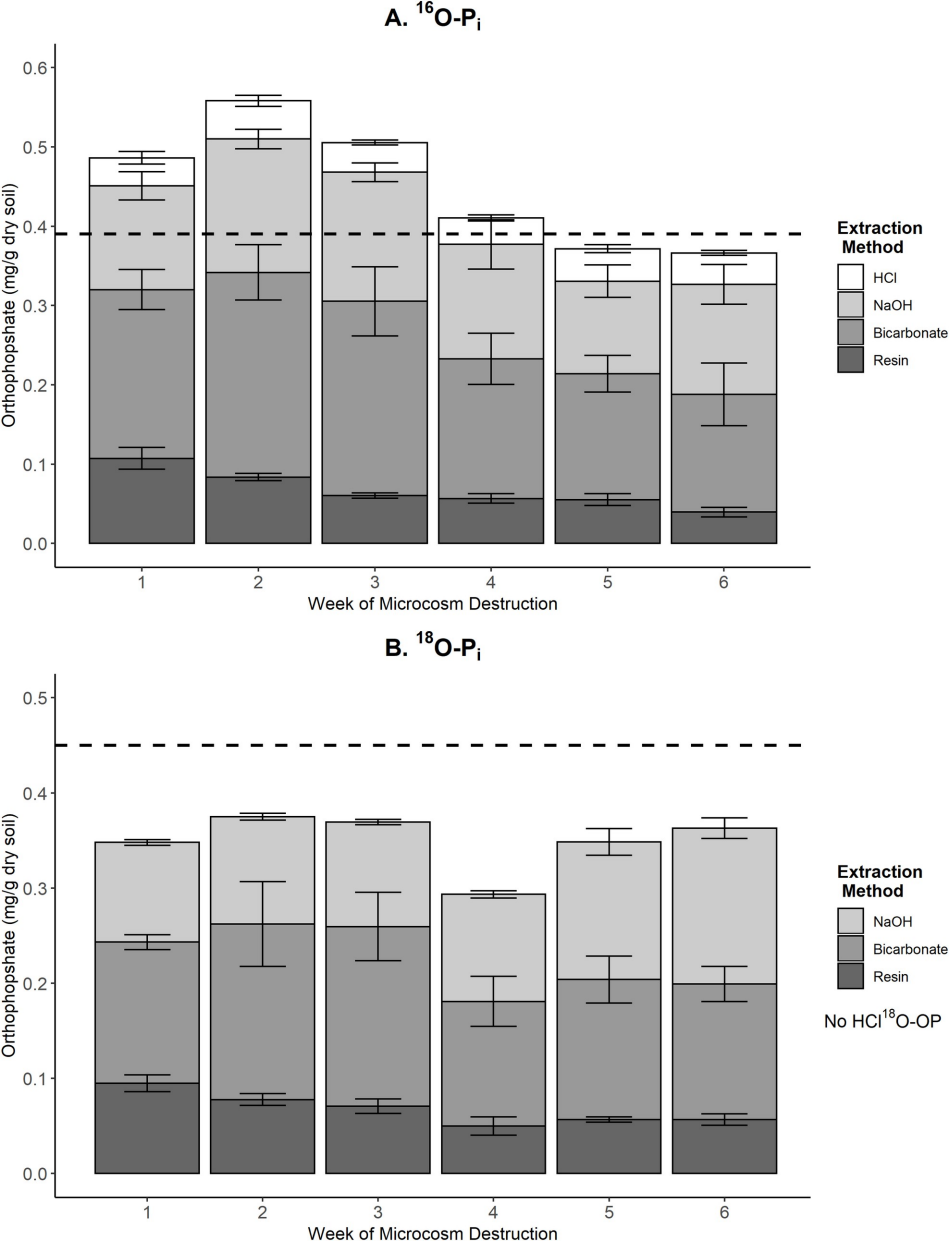
Mass balance of total P concentration from ^16^O-P_i_ and ^18^O-P_i_ doped microcosms by fractionation extraction. Each bar represents the average of 4 microcosms for the extraction methods (resin strips, bicarbonate, NaOH, HCl), with error bars indicating the standard error of the estimate. (A) Total ^16^O-PI from ^16^O- P_i_ doped microcosms. The dashed line represents ^16^O- P_i_ doped into the microcosms (0.39 mg/g dry soil = 0.066 mmol PI). (B) Total ^18^O- P_i_ from ^18^O- P_i_ doped microcosms measured on the 4000 QTRAP. The dashed line represents ^18^O- P_i_ doped into the microcosms (0.45 mg/g dry soil = 0.07 mmol P_i_). No ^18^O-HCl was measured in the ^18^O- P_i_ doped microcosms.

The total concentration of P_i_ during the time series varied by week, treatment, and extraction method (Fig 3). However, the amount of endogeous P_i_ strongly influenced the quantity of total ^16^O- P_i_ during the time series (Fig 3A). The weekly mean of ^16^O- P_i_ fluctuated from 0.37 to 0.56 mg/g dry soil and percent recovery varied from 94 to 144% (Table 3). As the experimental soil was inconsistently homogenized using the Japanese slabcake method before addition into the experimental units, the spatial variation in endogenous P within the experimental soils caused large disparity in mean and percent recovery of ^16^O- P_i_ by week. In comparison, the average total concentration of ^18^O- P_i_ by week shared similarity during the time series(Fig 3B). The mean of ^18^O- P_i_ varies from 0.30 to 0.38 mg/g dry soil with a percent recovery ranging from 67 to 85%. These percent recoveries demonstrated that a substantial quantity of the doped ^18^O- P_i_ was recovered during sequential fractionation. Any ^18^O- P_i_ loss may be attributed to: not homogenizing the soil properly following microcosm destruction, incomplete extraction during sequential fractionation and isotope exchange between labelled biomarkers and unlabelled water by microorganisms. Overall, there was less dissimilarity in mean and percent recovery in ^18^O- P_i_ doped microcosms in comparison to ^16^O- P_i_ doped microcosms. Additionally, the SE of the mean of ^18^O- P_i_ doped microcosms were smaller than those of ^16^O- P_i_ doped microcosms. As experimental addition was the sole source of ^18^O- P_i_ into experimental units, a stronger ^18^O- P_i_ percent recovery was expected. Therefore, tracking the movement of ^18^O- P_i_ fertilizer produced robust information into experimental P movement over time relative to ^16^O- P_i_ analyses.

**Table 3:** Mean, standard error, and percent recovery of P_i_ by treatment.

Week of Extraction	^16^O-P_i_			^18^O-P_i_		
	Mean	SE	PR	Mean	SE	PR
	——mg/g dry soil——	%	——mg/g dry soil—	%
1	0.49	0.034	125.3	0.34	0.005	77.0
2	0.56	0.044	143.8	0.38	0.051	84.7
3	0.51	0.032	130.3	0.37	0.021	82.6
4	0.41	0.067	105.8	0.30	0.025	66.6
5	0.37	0.030	95.8	0.35	0.018	77.7
6	0.37	0.059	94.4	0.37	0.021	81.9

The treatment of ^16^O- P_i_ added 0.39 mg/g dry soil whereas the ^18^O- P_i_ treatment added 0.45 mg/g dry soil to each applicable microcosm. Means represent the average of 4 microcosms by week of destruction. The percent recovery represents the P_i_ mean divided by P_i_ concentration added by treatment.

SE,standard error; PR, percent recovery ^16^O- P_i_; ^16^O-orthophosphate; ^18^O- P_i_, ^18^O-orthophosphate.

## Discussion

### Benefits of methodology

In this study, we successfully tracked the movement of experimental ^18^O- P_i_ using a novel mass spectrometry (MS) method. This methodology improves prior efforts to analyze ^18^O- P_i_ by achieving absolute quantification of P from multiple soil pools using tandem mass spectrometry (MS/MS). Previous manuscripts focused on relative quantification of either pure samples or on a single P_i_ soil fraction, losing important insight into the movement of P in soils [29,38,45,49]. Absolute quantification allowed the creation of an ^18^O- P_i_ mass balance to examine P pool movement and development over the time series, a unique feature to this study. While the MS and AA3 measured similar resin-extractable P_i_ results, the MS is a more robust instrument as it differentiates between ^16^O and ^18^O atoms. Additionally, the use of MS/MS provides significant benefits over ^18^O- P_i_ studies that used single quadrupole instruments [29,38,45,49]. In comparison to MS/MS, single quadrupoles have lower selectivity due to interference from co-eluting compounds and matrices [50]. This is essential as the Hedley sequential extraction matrices have a negative effect on the LLOQs due to high salt concentrations, where measured limits varied from 0.075 mg/L for 0.5 M HCl to 0.3 mg/L for 0.5 M bicarbonate. Newer triple quadrupole instruments have the capacity to achieve greater sensitivity and selectivity into picogram/mL range [51], which will aid to decrease the LLOQ. Furthermore, the use of MS/MS allows for improved accuracy and reproducibility at the lower end of the calibration curve [50], permitting examination of P-deficient soils. Focusing on each dNMP of DNA in an ^18^O- P_i_ study is a distinct characteristic of our procedure to measure the organic P pool. Previous studies concentrated on a single dNMP (dTMP), and were unable to monitor the 3 other dNMPs present in DNA [52].

### Geochemical and biological Phosphorus trends

The precipitation, adsorption-desorption, and biological effects of the PI fertilizers are like previous Hedley fractionation studies (Figs 2 and S1). Similar to our results, as P_i_ declined in the resin extractable pool, the concentration of bicarbonate P_i_ increased [53]. In agreement with both Qian and Schoenau [54] and Wagar et al. [55], we report that bicarbonate P_i_ represents the largest proportion extracted following fertilizer application. Short term studies often demonstrate a slight increase in P within recalcitrant fractions, where solubility decreases as P geochemically fixes to Ca-phosphates [10,56]. The ^18^O- P_i_ NaOH fraction concentration increased overtime from more labile pools; however, this was not apparent in ^16^O- P_i_ NaOH pool. As ^18^O- P_i_ is not naturally occurring, the short term experiment provided greater sensitivity into the movement of labelled fertilizer to more recalcitrant fractions. Finally, the absence of fertilizer P movement to the HCl pools agrees with Helfenstein et al. [57] where the development of HCl-extractable P takes years to centuries to form.

Our study reveals that isotopic composition does not influence P movement; however, previous studies are divided on whether labelled PI influences geochemical and biological processes. The labile fractions results are in agreement with previous studies that reported the sorption of ^16^O- and ^18^O- P_i_ to synthetic ferrihydrite reached equilibrium after 20 hours under abiotic conditions [58]. Although, our findings are in disagreement with Melby et al. [29], which reported that multiple ^18^O atoms within P_i_ causes greater sorption to soils. Moreover, the shared trends from resin-extractable and bicarbonate P extractions suggests that nutrient uptake by microbial communities is likely not influenced by isotopic composition of P_i_. This is in contrast to results stating that microbial communities prefer lighter isotopologues [59]. Our study outcomes are consistent with Mamet et al. [24] who reported that microorganisms do not have a preference for P_i_ by isotopic composition. While the MS measured resin-extractable ^18^O- P_i_ after 6 weeks, others found that the concentration of the labelled species becomes negligible after 50 days in aerobic non-sterilized soils [60]. Conflicting results may be attributed to anaerobic versus aerobic conditions as biological activity is much greater in the presence of O, producing a higher microbial P_i_ uptake [61]. Alternatively, Melby et al. [29] did not consider the movement of ^18^O-P_i_ to other pools of P within the soil system.

### Trends in NaOH and DNA P_i_

The small concentration of P_i_ from dNMP, one of the largest pools of organic P [62], signifies that the majority of the NaOH pool is in inorganic forms of P, specifically Fe and Al species [46]. Nevertheless, NaOH and dNMP results displayed the greatest fluctuations over time and rapid ^18^O- P_i_ delabelling compared to the other fractions; however, a small amount of labelled DNA remained within the macromolecule. Microorganisms negatively impacted DNA labelling as the greatest period of activity in ^18^O- P_i_ microcosms synthesized a small concentration of ^18^O-dNMP. The very small concentration ^18^O-labelled DNA is in agreement with previous studies that found that biotic systems rapidly exchange isotopes between P_i_ and water [27,63]. Previous studies established that dNMP labels quickly following incubation in H_2_^18^O doped soil [52,64]. However, as our study focuses on ^18^O- P_i_ uptake by microorganisms, the amount of time required to incorporate the isotopically labelled substrates will differ from H_2_^18^O studies. Future work into organic P movement requires consideration into the species not measured by the NaOH fraction, such as organic matter isolated by the labile-resin and bicarbonate fraction [5].

### Comparison of 32/33P to 18O-P_i_ mass spectrometry

The absence of ^18^O- P_i_ in the HCl extraction fractions after 6 weeks confirmed the radioisotopes ^32^P and ^33^P are incapable of offering an appropriate experimental length to follow P fertilizers. Measuring the suitable kinetic equilibrium time to produce recalcitrant ^18^O- P_i_ minerals may not be conducted using ^32/33^P as natural decay limits analysis to a few months [65]. The abiotic stability ^18^O- P_i_ provides the availability of longer experimental times to follow the fate of fertilizer to inaccessible forms of P minerals. Furthermore, we were able to decipher temporal movement of biotic activity from the concentration of dNMP from DNA; a task not possible with radioisotopes.

### Comparison of δ18O to 18O-P_i_ mass spectrometry

The limited sample preparation and the capability for absolute quantification favours HLPC-MS/MS measurement of ^18^O- P_i_ over δ^18^O to facilitate examination of P dynamics. In comparison to IRMS, ESI is a soft ionization MS technique that generates minimal fragmentation to the gas phase molecule, allowing for structural information [66]. Soils require substantial δ^18^O characterization as isotopic values vary both temporally and spatially; therefore, individual sources of P_i_ within each soil will possess unique signatures [67]. Co-eluting anions, such as nitrates and sulfates, and ions, like Na^+^ and Cl^-^, interfere with δ^18^O analysis in P_i_ by IRMS [49,68]. While the use of Ag_3_PO_4_ is considered the most suitable standard for ^18^O measurement, there are current no certified standards [69]. Alternatively, P_i_ retains its shape during MS/MS quantification, as the instrument examines the mass to charge ratio of gas phase ions prior to and after the collision cell. Soil samples for MS analysis do not require background characterization as ^18^O- P_i_ was absent from both the ^16^O- P_i_ doped and control microcosms here and in previous studies [60]. The MS directly measures the concentration of P_i_ by using calibration curves for both ^16^O- P_i_ and ^18^O- P_i_. While the isotopic forms of P_i_ co-elute, both may be used for MS/MS quantification as the species will not suppress the response of the analytes [70]. Mass spectrometry instruments do not affect P_i_ labelling as Alvarez et al. [49] reported that O exchange within phosphate species did not occur during MS quantification. Moreover, a quantifiable amount of naturally occurring ^18^O- P_i_ is unlikely to occur due to low environmental abundance [59]. Therefore, replacing current δ^18^O techniques with measuring P_i_ using MS will circumvent inconsistencies with quantification of the isotopically labelled substrates movement within soil ecosystems.

### Sample clean-up

While the methodology for measuring experimental ^18^O-P_i_ is applicable to all soil types, samples require cleanup prior to quantification on the MS/MS to remove excess salts from extraction solutions. Excessive salts interfere with detection and ionization by causing ion suppression [71]. Isolation of the respective P pools uses bicarbonate and NaOH solutions resulting in high sodium content and high pH. Our study sample preparations used resin beads to replace Na^+^ with H^+^, effectively lowering the concentration of salts and pH simultaneously. MS/MS requires lower pH to allow for protonation of gas phase ions [72]. For soils higher in Al and Fe, the Bray-1 and Mehlich-3 P extraction methods also generate a high volume of salts [73,74]. Resin beads can replace major cations and anions with H^+^ and OH^-^ ions. Another option to overcome ion suppression is chromatographic separation; however, this will require longer chromatographic runs for sample and column clean-up [71]. Overall, proper sample preparation for MS/MS measurement of ^18^O- P_i_ allows the methodology to become available for all soil types to better understand the P cycle.

## Conclusions

This document presents a MS method that improves current ^18^O-isotope analysis to define inorganic and organic P cycling within soils. This protocol is accessible for all soil types; however, MS requires specific sample preparation to remove excess ions that inhibit ionization. Biological techniques such as SIP can use this method to verify isotopic incorporation into isopycnically separated DNA. While the purpose of this manuscript was to present the methodology, we found potential to provide new information in long-term P soil dynamics from the absence of ^18^O- P_i_ in the HCl fraction. Future prospects of interpreting P dynamics using the ^18^O- P_i_ MS method include the combination of spectroscopic and isotopic techniques as well as the combined use of radioisotopes ^32/33^P with ^18^O to understand P fertilizer in soils [57]. The method we have outlined here provides new opportunities to resolve broken links in the P cycle.

## Supporting information

S1 Fig Total temporal change in ^16^Oxygen-orthophosphate and ^18^Oxygen-orthophosphate in microcosms extracted by sequential fractionation. Each symbol represents the average of 4 microcosms, with error bars indicating the standard error of the estimate. (A) Quantification of ^**16**^**Oxygen-orthophosphate** from ^**16**^**Oxygen-orthophosphate** doped microcosms. (B) Quantification of ^**16**^**Oxygen-orthophosphate** from ^**18**^**Oxygen-orthophosphate** doped microcosms. (C) Quantification of ^**18**^**Oxygen-orthophosphate** from ^**18**^**Oxygen-orthophosphate** doped microcosms.
(DOCX)

S1 Table QTRAP 4000 parameters for the optimization of ^16^Oxygen-orthophosphate and ^18^Oxygen-orthophosphate and the deoxynucleoside monophosphate isotopologues. (DOCX)

S2 Table Chromatographic and QTRAP 4000 parameters for the quantification of ^16^Oxygen-orthophosphate and ^18^Oxygen-orthophosphate. (DOCX)

S3 Table Chromatographic and QTRAP 4000 parameters for the quantification of the deoxynucleoside monophosphate isotopologues. (DOCX)

S1 Data (XLSX)
