# Supplementary material for: Methodology and validation of a new tandem mass spectrometer method for the quantification of inorganic and organic 18O-phosphate species
Source: PLoS One. 2020 Feb 24;15(2):e0229172. doi: 10.1371/journal.pone.0229172 (PMC7039501; doi:10.1371/journal.pone.0229172)
Supplement: S1 Fig — Each symbol represents the average of 4 microcosms, with error bars indicating the standard error of the estimate. (A) Quantification of 16Oxygen-orthophosphate from 16Oxygen-orthophosphate doped microcosms. (B) Quantification of 16Oxygen-orthophosphate from 18Oxygen-orthophosphate doped microcosms. (C) Quantification of 18Oxygen-orthophosphate from 18Oxygen-orthophosphate doped microcosms. (DOCX) [file pone.0229172.s001.docx]

**SUPPLEMENTAL FIGURE**


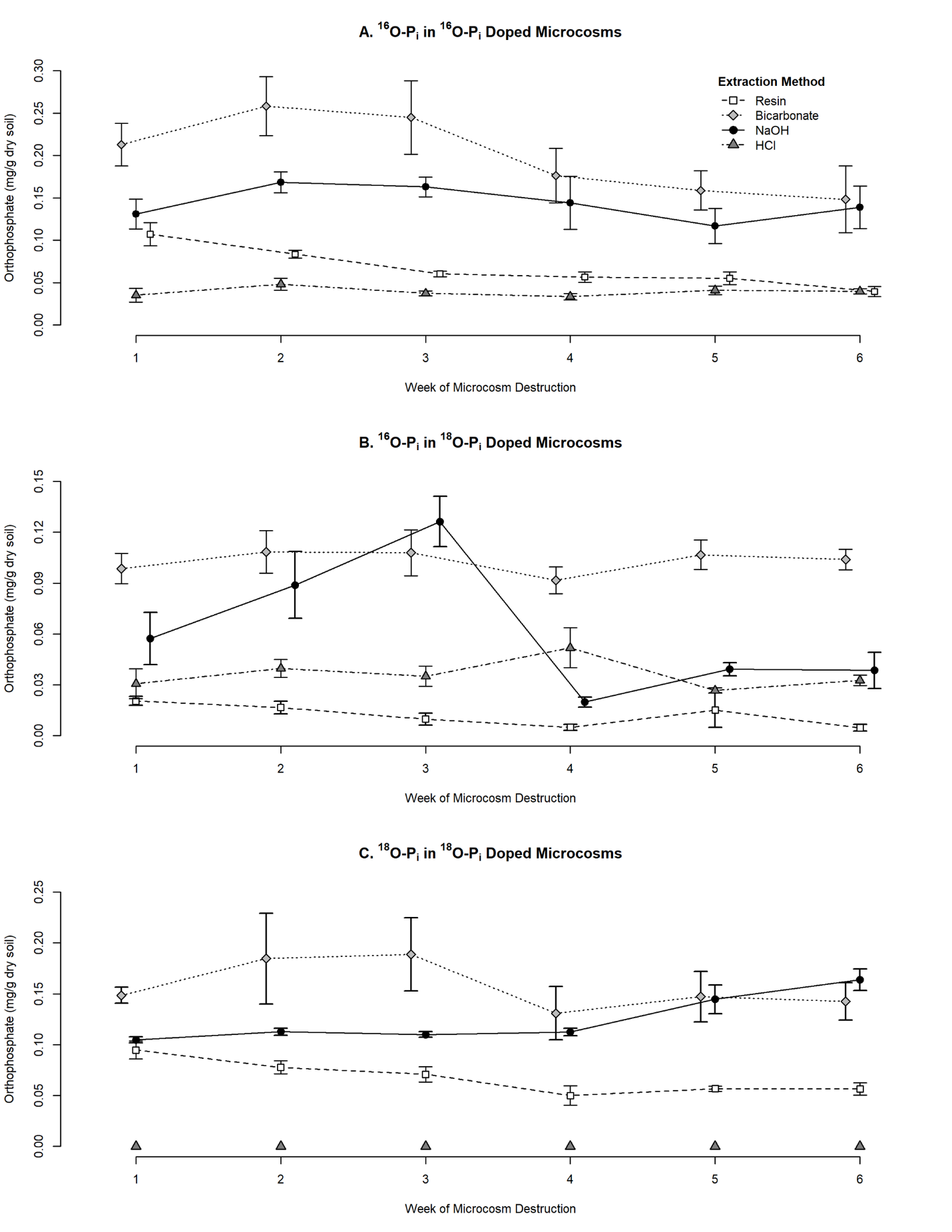


**Supplemental FIG 1:** Quantification of ^16^O‑P_i_ and ^18^O‑P_i_ (mg/g dry soil) from doped microcosms via sequential fractionation extraction versus week of microcosm destruction. Each symbol represents the average of 4 microcosms, with error bars indicating the standard error of the estimate. A) Quantification of ^16^O‑P_i_ from ^16^O‑P_i_ doped microcosms. B) Quantification of ^16^O‑P_i_ from ^18^O‑P_i_ doped microcosms. C) Quantification of ^18^O‑P_i_ from ^18^O‑P_i_ doped microcosms.
