# Supplementary material for: Methodology and validation of a new tandem mass spectrometer method for the quantification of inorganic and organic 18O-phosphate species
Source: PLoS One. 2020 Feb 24;15(2):e0229172. doi: 10.1371/journal.pone.0229172 (PMC7039501; doi:10.1371/journal.pone.0229172)
Supplement: S1 Table — (DOCX) [file pone.0229172.s002.docx]

**Supplementary Table 1:** QTRAP 4000 parameters for the optimization of ^16^Oxygen-orthophosphate and ^18^Oxygen-orthophosphate and the deoxynucleoside monophosphate isotopologues.

| **Instrument Parameter** | **Value** |
| --- | --- |
| Polarity | Negative |
| Infusion Rate (µL/min) | 10 |
| Curtain Gas (psi) | 45 |
| Collision Gas (psi) | 6 |
| Collision Energy (V) | -20 to -5 |
| Declustering Potential (V) | -40 |
| Ion Spray Voltage (V) | -4500 |
| Interface Temperature (°C) | 500 |
| Ion Source Gas 1 (psi) | 45 |
| Drying Gas and ESI^‡^ Nebulizing Gas | Nitrogen |

PSI, pounds per square inch; ESI, Electrospray Ionization; V, volts.
