# Supplementary material for: Methodology and validation of a new tandem mass spectrometer method for the quantification of inorganic and organic 18O-phosphate species
Source: PLoS One. 2020 Feb 24;15(2):e0229172. doi: 10.1371/journal.pone.0229172 (PMC7039501; doi:10.1371/journal.pone.0229172)
Supplement: S2 Table — (DOCX) [file pone.0229172.s003.docx]

**Supplementary Table 2:** Chromatographic and QTRAP 4000 parameters for the quantification of ^16^Oxygen-orthophosphate and ^18^Oxygen-orthophosphate.

| **Instrument Parameter** | **Value** |
| --- | --- |
| Column | None |
| Mobile Phase (A) | 0.1% Formic Acid in Water |
| Mobile Phase (B) | 0.1% Formic Acid in Methanol |
| Elution Method | Isocratic: 50:50 A/B |
| Run Time (min) | 5 |
| Flow Rate (mL/min) | 0.2 |
| Injection Volume (µL) | 5 |
| Polarity | Negative |
| Curtain Gas (psi) | 45 |
| Collision Gas (psi) | 6 |
| Ion Spray Voltage (V) | -4500 |
| Interface Temperature (°C) | 500 |
| Ion Source Gas 1 (psi) | 45 |
| Ion Source Gas 2 (psi) | 40 |
| Collision-Activated Dissociation Gas | High |

PSI, pounds per square inch; V, volts.
