# Supplementary material for: Methodology and validation of a new tandem mass spectrometer method for the quantification of inorganic and organic 18O-phosphate species
Source: PLoS One. 2020 Feb 24;15(2):e0229172. doi: 10.1371/journal.pone.0229172 (PMC7039501; doi:10.1371/journal.pone.0229172)
Supplement: S3 Table — (DOCX) [file pone.0229172.s004.docx]

**Supplementary Table 3:** Chromatographic and QTRAP 4000 parameters for the quantification of the deoxynucleoside monophosphate isotopologues.

| **Instrument Parameter** | **Value** |
| --- | --- |
| Column | Zorbax Eclipse XDB-C18 4.6 x150 5μ column (Agilent Technologies) |
| Column Temperature (°C) | 40 |
| Mobile Phase (A) | 0.1% Formic Acid in Water |
| Mobile Phase (B) | 10 mM Ammonium Acetate in Methanol |
| Elution Method | 100% A for 10 min at 0.1 mL/min  0 to 20% B to 14 min at 0.1 mL/min  100% B to 15 min at 0.1 mL/min  100% B to 30 min at 1.0 mL/min  100 % A to 32 min at 0.1 mL/min |
| Divert Flow Instrument | Valco valve (VICI - Valco Instruments Co. Inc, Houston TX, USA) |
| Run Time (min) | 32 |
| Sampling Time (min) | 12 to 20 (diverted to waste from 0 to 12 and 20 to 32) |
| Flow Rate (mL/min) | Variable |
| Injection Volume (µL) | 35 |
| Polarity | Negative |
| Curtain Gas (psi) | 15 |
| Collision Gas (psi) | 6 |
| Ion Spray Voltage (V) | -4500 |
| Interface Temperature (°C) | 500 |
| Ion Source Gas 1 (psi) | 65 |
| Ion Source Gas 2 (psi) | 55 |
| Collision-Activated Dissociation Gas | High |

PSI, Pounds per square inch
